# Systemic Bacillus Calmette-Guérin (BCG) Infection With Renal Involvement: A Rare Complication of BCG Immunotherapy

**DOI:** 10.7759/cureus.33134

**Published:** 2022-12-30

**Authors:** Raquel Afonso, Joana Fontes, Pedro Pinto, Miguel Romano, Alexandra Esteves

**Affiliations:** 1 Internal Medicine, Hospital de Santa Luzia, Viana do Castelo, PRT; 2 Internal Medicine, Hospital Conde de Bertiandos, Viana do Castelo, PRT; 3 School of Medicine, Minho University, Braga, PRT

**Keywords:** mycobacterium bovis, bladder cancer, renal abscess, systemic bcg infection, bacillus calmette-guérin (bcg) immunotherapy

## Abstract

Intravesical instillation of bacillus Calmette-Guérin (BCG) is the adjuvant therapy for superficial urothelial carcinoma of the bladder with the lowest recurrence rates and is well tolerated with minor and self-limiting adverse effects. Serious complications, such as systemic BCG infection, are uncommon as the diagnosis is difficult and, in the majority of cases, *Mycobacterium bovis* cannot be isolated.

We describe a case of a man who presented with prolonged fever associated with polyuria, dysuria, anorexia, and significant weight loss, refractory to several courses of appropriate antibiotic therapy. After an exhaustive investigation, the underlying diagnosis of systemic BCG infection with renal involvement was considered. Antituberculosis treatment resulted in a marked clinical and radiological recovery, supporting this diagnosis.

## Introduction

Intravesical instillation of bacillus Calmettee-Guérin (BCG), a live attenuated strain of *Mycobacterium bovis*, remains the standard of care in patients with high-risk superficial bladder cancer, being the most commonly used and the most effective intravesical agent for this malignancy and being associated with increased disease-free survival and lower rates of tumor recurrence and progression [1,2].

The effect of intravesical BCG on tumor progression and recurrence prevention is not fully understood [3], although it appears to be related to an immunomodulatory process where local immune activation leads to the local migration of polymorphonuclear cells and the subsequent immune destruction of tumor cells [4,5].

BCG therapy is usually well-tolerated, with some minor side effects reported such as cystitis, hematuria, and fever [2,6]. Serious adverse events are uncommon (<5%) and are usually related to systemic BCG infection (less than 1%) [7].

As microbiological studies are usually negative, the diagnosis is challenging and requires a high index of suspicion [3,8-10]. Sepsis is its most serious manifestation, but infection of several organs, such the liver, bone, and lung, can occur [3,8-10]. Renal involvement is rare (0.3%-3.5% of cases), usually presenting as a renal abscess or nephritis [11].

## Case presentation

A 77-year-old male, with a history of arterial hypertension, dyslipidemia, and type 2 diabetes mellitus, was diagnosed with a high-grade bladder urothelial carcinoma, without muscular invasion (pT1), after performing a transurethral resection of the tumor. The patient initiated a schedule of immunotherapy with BCG (induction: once weekly for six weeks; maintenance: once weekly for three weeks performed every three, six, 12, 18, 24, 30, and 36 months), without major adverse reactions except flu-like symptoms lasting more than 48 hours after each instillation.

Following the eighth instillation, he presented with fever (maximum: 38.5°C), urinary urgency, and dysuria for more than 48 hours of evolution. He was admitted to the emergency department and later discharged with a diagnosis of cystitis, treated with norfloxacin 400 mg/day for seven days. Following two weeks of persistent evening fever and urinary complaints (urinary urgency and dysuria) associated with worsening asthenia and anorexia, he was readmitted to the emergency department. The physical observation was unremarkable. Urine analysis showed pyuria (23 leukocytes per field). Although the laboratory workup revealed acute kidney injury (creatinine: 1.66 mg/dL) and elevated C-reactive protein (10.69 mg/dL), he was discharged with a prescription of cefuroxime 500 mg 12/12 hours.

**Figure 1:**
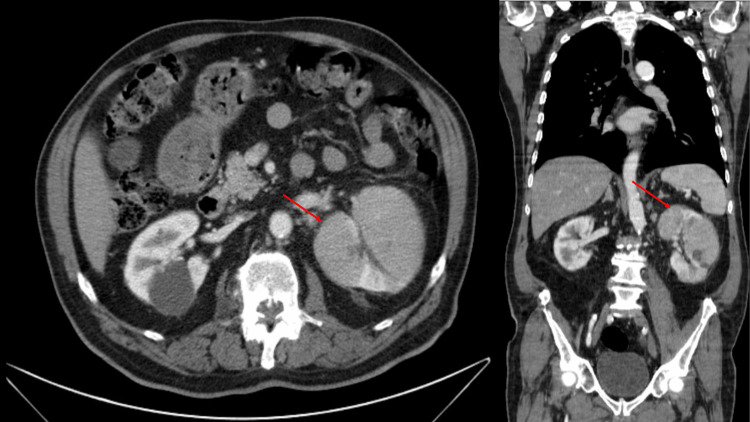
Admission abdominopelvic scan showing globosity of the left kidney with areas of heterogeneity (arrow).

On the fifth day of antibiotic therapy, the patient was again readmitted to the hospital with persistent evening fever (maximum: 38.5°C), left flank pain, and a quantified weight loss of 12 kg in six weeks but with resolution of urinary complaints. On admission, he was febrile (38.4°C) and hypotensive (88/43 mmHg). The remaining physical examination presented no alterations. Laboratory tests revealed increased levels of C-reactive protein (11.95 mg/dL), kidney dysfunction (creatinine: 1.57 mg/dL), hematuria, and leukocyturia. Abdominal, renal, and pelvic tomography revealed a globosity of the left kidney with areas of heterogeneity and perinephric fat densification, indicating possible pyelonephritic changes (Figure 1). Chest radiography showed no images suggestive of pulmonary infiltrate, cavitations, pleural effusion, or pneumothorax. He was admitted to the internal medicine infirmary and started on fluid therapy and piperacillin and tazobactam 4.5 g every eight hours.

As fever, nocturnal sweating, anorexia, and asthenia persisted after eight days of antibiotic therapy, further etiologic investigation was performed. Laboratory investigations revealed elevated erythrocyte sedimentation rate (61 mm/hour) and adenosine deaminase (ADA) levels (36.4 UI/L). The remainder of the laboratory results, including thyroid, hepatic, and renal function tests, was normal, as were electrolytes and glucose blood levels. HIV testing and hepatotropic viruses were also negative. Markers of immune-mediated disease (antinuclear antibodies, serum immunoglobulins, and complement levels) were negative. To exclude endocarditis, the patient had a transthoracic echocardiogram that showed no changes in the cardiac function and no masses or vegetation. Multiple cultural microbiological studies of urine, blood, and sputum were performed and revealed to be negative for both bacteria and mycobacteria. The detection of *Mycobacterium tuberculosis* complex by polymerase chain reaction (PCR) amplification testing in urine was positive, although the patient had no recent sick or tuberculosis contacts, and interferon-gamma release assay (IGRA) for *Mycobacterium tuberculosis* infection was negative.

Considering the presenting symptoms with indolent progression, absence of clinical improvement despite antibiotic treatment, presence of sterile pyuria, and detection of *Mycobacterium tuberculosis* complex in urine by PCR (possible relation to previous BCG treatment), the diagnosis of systemic BCG infection complicated with a renal abscess was suggested, and empiric antituberculosis treatment was started with isoniazid 300 mg, rifampin 600 mg, and ethambutol 1,200 mg once daily.

**Figure 2:**
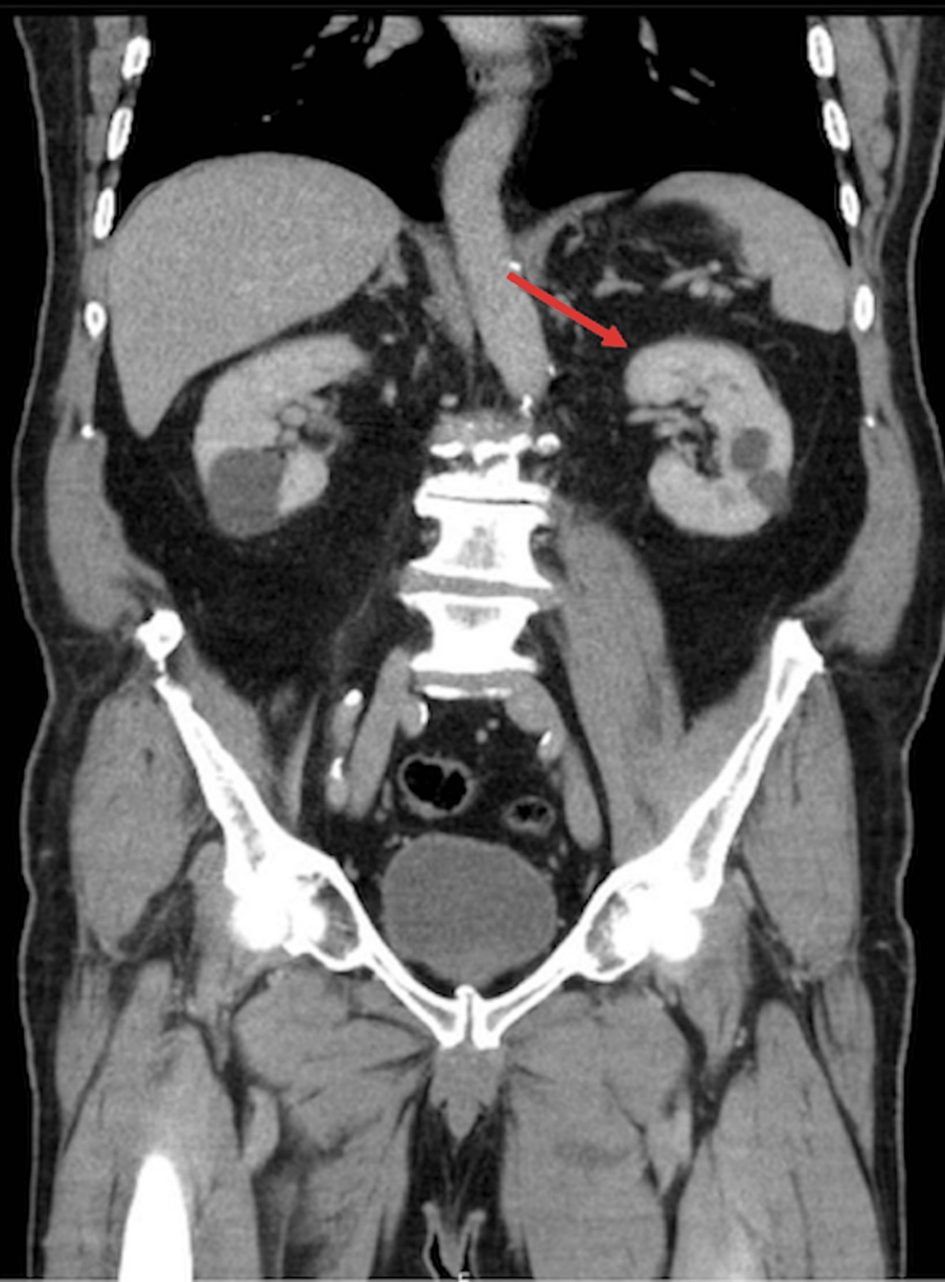
Follow-up abdominopelvic scan showing resolution of the renal changes: globosity of the left kidney with areas of heterogeneity (arrow).

With the initiation of the antituberculous therapy, the patient progressed favorably with resolution of symptoms (fever, nocturnal hypersudorrhea, anorexia, and asthenia). He was discharged home with complete recovery after 17 days of hospitalization. The patient was treated with anti-bacillary agents for six months and stopped immunotherapy with BCG. A renal tomography performed six months after discharge showed resolution of the renal changes described previously (Figure 2). No signs of relapse were observed after 11 months of follow-up.

## Discussion

Although BCG intravesical instillation usually exhibits a favorable safety profile, since it contains viable attenuated mycobacteria, the potential for serious adverse events exists [4].

Complications of this treatment that were described in the literature include prostatitis, epididymo-orchitis, balanitis, ureteral obstruction, bladder contraction, persistent symptomatic mycobacteriuria, severe sepsis, other orthopedic, rheumatologic, pulmonary, hepatic, renal, and vascular complications, and severe sepsis [3,12].

Concerning the risk of systemic invasion, an absolute contraindication is BCG instillation immediately after transurethral resection or an overt traumatic catheterization. Additionally, patients with fever and urinary tract infections should have their treatment postponed [13]. Relative contraindications reported are conditions related to immunological compromise, such as the use of immunosuppressive agents and HIV infection [13].

Systemic BCG infection should be suspected in a patient who develops moderate to severe genitourinary or systemic symptoms following one or more instillation of intravesical BCG, with no adequate alternative diagnosis and with a favorable response to antituberculosis treatment. The cornerstones of the diagnosis of systemic BCG infection are; therefore, clinical and microbiological or histopathologic evidence of mycobacterial infection is not necessary as serologic tests and cultures are negative in approximately 60% of cases [2]. This patient had a positive detection of *Mycobacterium tuberculosis* complex in urine by PCR. Although favorable to the diagnosis, this result could be related to previous BCG instillations, reflecting the amplification of DNA from nonviable mycobacteria, and should not be considered as definitive diagnostic criteria.

As previously stated, the risk factors for systemic BCG infection include states of immunosuppression, but old age and a greater extent of bladder mucosal damage from the tumor are also known to increase its risk [1,14]. Our patient was diabetic, which gives him some degree of immunosuppression, and he was over 70 years old.

The optimal treatment for systemic BCG infection is uncertain but is mainly based on case series and clinical experience, taking into account the clinical presentation and severity of symptoms [9]. Several case series in the literature suggest the combination of antimycobacterial therapy and corticosteroids as the pathogenesis of the systemic infection can include a combination of the infectious potential of the mycobacteria allied to a local inflammation with a hypersensitivity reaction, which could explain the negative results of cultures and serological tests in highly symptomatic patients, and their favorable response to steroids [9].

In our case, the patient’s symptoms were probably due to an active BCG infection, as its favorable clinical response was achieved with anti-bacillary agents alone.

As *Mycobacterium bovis* is resistant to pyrazinamide, the first-line treatment includes rifampin, isoniazid, and ethambutol for a total treatment duration of six months [2]. In cases of extensive miliary involvement, respiratory failure, and clinical signs of hypersensitivity reactions such as pneumonitis and hepatitis, corticosteroid therapy should be considered, although no standardized regime has been recommended [14].

The prognosis of systemic infection is favorable when treatment is started early in the course of the disease [3], although 5.4% of mortality rate has been reported, mostly due to respiratory, liver, and multiple organ failure [13]. Our patient had a very favorable clinical response, with complete recovery to date.

We highlight the case as it is a rare complication of BCG treatment, as well as the importance of clinical suspicion of systemic BCG infection in cases of sterile pyuria, especially in patients undergoing BCG immunotherapy.

## Conclusions

Systemic BCG infection is a rare and serious complication of intravesical BCG immunotherapy. The diagnosis is challenging and is based mostly on clinical history since the microbiological study is most often negative, and high clinical suspicion is required. The recommended treatment is the combination of antituberculosis agents with steroids, which when started early are associated with a good prognosis.
